# A Diagnostic Dilemma: Daptomycin-Induced Drug Reaction With Eosinophilia and Systemic Symptoms (DRESS) Syndrome and Eosinophilic Pneumonia With Concurrent COVID-19

**DOI:** 10.7759/cureus.77736

**Published:** 2025-01-20

**Authors:** Husam Shakour, Reem Mumtaz, Abdel-Ghanie Abu-Samra

**Affiliations:** 1 Internal Medicine, University of Kansas School of Medicine Wichita, Wichita, USA; 2 Pulmonology/Critical Care, University of Kansas School of Medicine Wichita, Wichita, USA

**Keywords:** acute hypoxemic respiratory failure, covid 19, daptomycin induced pneumonitis, drug reaction with eosinophilia and systemic symptoms (dress) syndrome, lekocytoclastic vasculitis

## Abstract

Adverse drug reactions are critical considerations in managing complex medical conditions. Drug reaction with eosinophilia and systemic symptoms (DRESS) syndrome is a rare but potentially life-threatening hypersensitivity reaction characterized by rash, eosinophilia, and multisystem involvement. Daptomycin, an antibiotic commonly used for resistant Gram-positive infections, has been associated with both eosinophilic pneumonia and DRESS syndrome. Challenges in diagnosing daptomycin-induced eosinophilic pneumonia arise due to overlapping symptoms with infectious or inflammatory processes, further complicated by concurrent COVID-19 infection. This case highlights a patient with acute hypoxemic respiratory failure (AHRF) attributed to DRESS syndrome caused by daptomycin, underscoring the diagnostic challenges and therapeutic considerations in such scenarios.

## Introduction

Drug-induced hypersensitivity reactions are uncommon but clinically significant complications that demand prompt recognition and management. Drug reaction with eosinophilia and systemic symptoms (DRESS) syndrome and eosinophilic pneumonia are rare adverse effects of daptomycin, an antibiotic commonly used for resistant Gram-positive infections [1]. The overlapping clinical presentation of drug-induced reactions and infectious diseases, such as COVID-19, can complicate diagnosis and delay appropriate treatment. Here, we present a case of a 39-year-old female with acute hypoxemic respiratory failure (AHRF) due to DRESS syndrome and eosinophilic pneumonia caused by daptomycin, with concurrent COVID-19 infection.

## Case presentation

**Figure 1:**
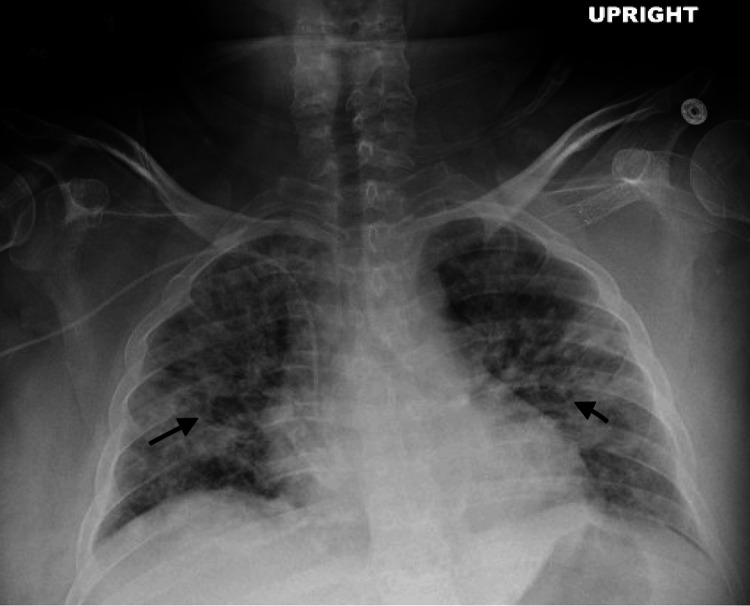
New onset bilateral alveolar infiltrates compared to previous imaging

**Figure 2:**
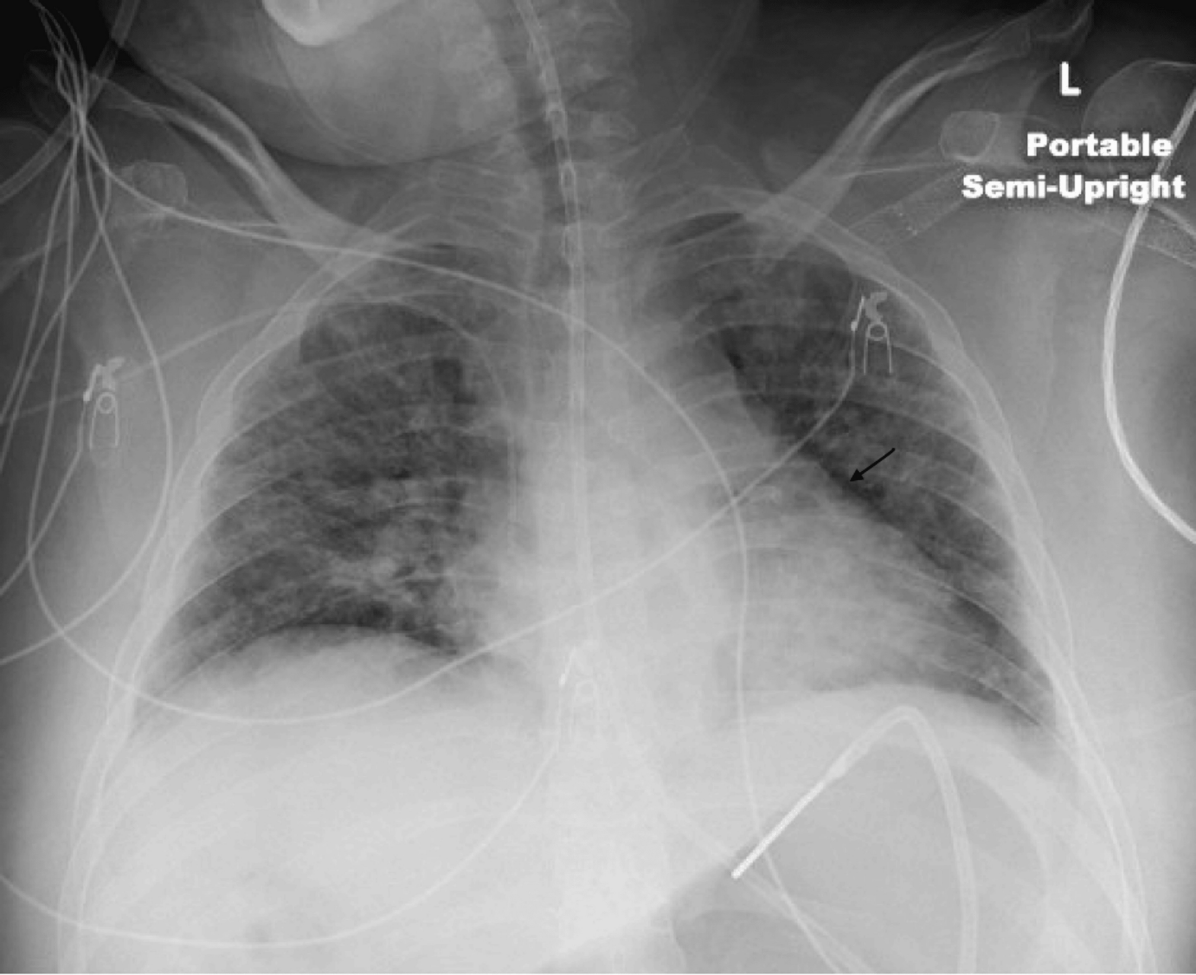
Improved bilateral alveolar infiltrates compared to Figure 1

A 39-year-old female with a history of uncontrolled type 1 diabetes mellitus, end-stage renal disease (ESRD) status post kidney transplant two years prior, and peripheral arterial disease was hospitalized for left foot osteomyelitis, requiring transmetatarsal amputation. At hospitalization, she started vancomycin for 12 days, later switching to daptomycin for methicillin-resistant Staphylococcus aureus (MRSA) positive wound cultures. Her course was complicated by a new rash initially attributed to a dose of Lyrica for neuropathic pain. Biopsy revealed leukocytoclastic vasculitis wherein she was treated with prednisone. After stabilizing clinically, she was discharged on prednisone and daptomycin. Two days later, she returned with worsening lethargy, cough, hypoxemia requiring oxygen, and hypotension. Imaging revealed bilateral pulmonary infiltrates (Figure 1) and she tested positive for COVID-19. Her initial presentation was attributed to COVID pneumonitis and she was started on remdesivir. Despite these interventions, her clinical status deteriorated. On the second day of hospitalization, cefepime was added empirically for a possible bacterial superinfection. General immunologic workup, including C-antineutrophil cytoplasmic antibodies (ANCA), P-ANCA, antinuclear antibodies (ANA), anti-double stranded DNA (dsDNA), and myeloperoxidase (MPO)-ANCA, was negative. However, by the third day of her admission, her respiratory condition had worsened, necessitating intubation and mechanical ventilation due to severe AHRF. Hospitalization was also complicated by an acute kidney injury, prompting concerns for a systemic drug reaction. Given negative immunological workup and high suspicion of DRESS syndrome a peripheral eosinophil count was checked and was elevated at 1970 (was 0 a few days before daptomycin was started). DRESS syndrome was suspected and daptomycin was discontinued and exchanged with vancomycin in addition to high-dose methylprednisolone. These findings, combined with her multisystem involvement - rash with a biopsy showing leukocytoclastic vasculitis, renal dysfunction, and pulmonary infiltrates - led to the diagnosis of DRESS syndrome. Microbiologic workup, including blood and sputum cultures, was negative, further supporting a non-infectious etiology for her respiratory failure. Over the following days, her clinical condition improved. She was extubated after four days and transitioned to a steroid taper. Repeat imaging demonstrated improvement of the bilateral pulmonary infiltrates (Figure 2). 

## Discussion

Daptomycin has been associated with eosinophilic pneumonia and systemic hypersensitivity reactions, including DRESS syndrome [1,2]. Acute eosinophilic pneumonia results when alveolar macrophages detect an antigen, triggering recruitment of T-helper 2 lymphocytes. These lymphocytes release interleukin 5, which stimulates eosinophil production and migration to the lungs [3]. In this case, the patient's COVID-19 infection created a diagnostic bias, delaying the recognition of a drug-induced reaction. The overlapping symptoms of COVID pneumonitis, such as hypoxia, fever, and bilateral infiltrates, masked other potential causes of her acute hypoxemic respiratory failure. Clinical features of daptomycin-induced eosinophilic pneumonia include dyspnea, hypoxemia, fever, and diffuse infiltrates on imaging [4]. A bronchoalveolar lavage showing elevated eosinophils (>25%) is diagnostic although clinical improvement with drug discontinuation obviates the need for invasive testing [5]. This presentation was complicated by multisystem involvement, including renal dysfunction and biopsy-proven leukocytoclastic vasculitis, hallmark features of DRESS syndrome. Management focused on stopping daptomycin and starting high-dose corticosteroids, leading to rapid improvement. 

## Conclusions

The case underscores the importance of maintaining a broad differential diagnosis when managing patients with overlapping symptoms of infectious and non-infectious etiologies, particularly in the context of a concurrent COVID-19 infection. This report highlights the diagnostic challenges posed by drug-induced reactions, such as daptomycin-induced DRESS syndrome and eosinophilic pneumonia, which can mimic more common conditions like viral pneumonitis.

Early recognition and management of such adverse drug reactions are critical to improving patient outcomes, as evidenced by the rapid clinical improvement observed after discontinuation of daptomycin and initiation of high-dose corticosteroids. Clinicians should be alert to the potential for multisystem involvement, including skin manifestations, pulmonary infiltrates, and renal dysfunction, when evaluating cases with atypical clinical presentations.

The key takeaway from this case is that vigilance for adverse drug reactions, coupled with a systematic approach to differential diagnosis, is vital to avoid misattribution of symptoms and to ensure timely, effective intervention.
